# P20 The methodological quality of studies assessing healthcare students’ knowledge of antibiotic use and resistance: a systematic review

**DOI:** 10.1093/jacamr/dlaf118.027

**Published:** 2025-07-14

**Authors:** Asa Auta, Erick Wesley Hedima, Emmanuel O Adewuyi, Shalkur David, Emmanuel Agada David, Enoche Florence Oga, Davies Adeloye, Barry Strickland-Hodge

**Affiliations:** Faculty of Health, Social Care and Medicine, Edge Hill University, Ormskirk, L39 4QP, UK; Department of Clinical Pharmacy and Pharmacy Practice, Faculty of Pharmaceutical Sciences, Gombe State University, Gombe, Nigeria; School of Population Health, Faculty of Health Sciences, Curtin University, Perth, Western Australia, Australia; Department of Clinical Pharmacy and Pharmacy Practice, University of Jos, Jos, Nigeria; Department of Clinical Pharmacy and Pharmacy Practice, Faculty of Pharmaceutical Sciences, Gombe State University, Gombe, Nigeria; School of Pharmacy and Biomedical Sciences, University of Lancashire, Preston, UK; School of Health & Life Sciences, Teesside University, Middlesbrough Tees Valley, TS1 3BX, UK; School of Healthcare, University of Leeds, Leeds, UK

## Abstract

**Objectives:**

This systematic review assesses the methodological quality of studies investigating the knowledge of healthcare students on antibiotic use and resistance.

**Methods:**

The PubMed®, Embase® (via Ovid) and CINAHL (via EBSCO) databases were systematically searched for studies published between 01 January 2014 and 31 December 2024 that reported Healthcare students’ knowledge of antibiotic use and resistance. The quality of the studies was assessed using the Medical Education Research Quality Instrument (MERSQI), which comprises 10 items across six domains: study design, sampling, data type (subjective or objective), validity, data analysis, and outcomes. Each domain is scored up to a maximum of 3, resulting in a total possible MERSQI score of 18.

**Results:**

Of the 9165 articles identified, 119 studies with data from 46 countries met the inclusion criteria. The total MERSQI scores of the included studies ranged from 7.00 to 14.00, with a mean score of 10.51±1.46. The highest mean domain scores were for type of data (2.76 ± 0.65) and data analysis (2.71 ± 0.45). The lowest scores were for study design (1.01 ± 0.06) and validity (0.73 ± 0.78). Nearly all (98.3%) of the studies were single-group cross-sectional or post-test-only designs. Face validity, either explicitly stated or inferred, was indicated in 72 articles (60.5%), while content validity, ensuring items covered an appropriate range of topics, was reported in 54 articles (45.4%). Reliability statistics were provided in 31 studies (26.1%), with Cronbach’s α reported in 29 of them. The intraclass correlation coefficient (ICC) and Kuder-Richardson 20 (KR20) were each reported in a single study.

**Conclusions:**

Many of the reviewed studies were judged to have moderate methodological quality. Additionally, there was often a lack of information or clarity about the content validity of the instruments or questions used to evaluate antibiotic knowledge. The reliability of the study instruments, while extremely valuable, was not always determined or reported. Reporting reliability provides confidence that the results are dependable and not influenced by random errors.

